# Effect of Homoarginine on Coronary Artery Complexity and Atherosclerotic Burden in Patients with STEMI

**DOI:** 10.3390/jcm14051501

**Published:** 2025-02-24

**Authors:** Gülsüm Bingöl, Ahmad Huraıbat, Elif Ayduk Gövdeli, Özgür Selim Ser, Serkan Ünlü, Murat Çelik, Leyla Bulut, Özge Özden, Emre Özmen, Kadriye Kılıçkesmez

**Affiliations:** 1 Department of Cardiology, Arel University Medicine Faculty, 34010 Istanbul, Turkey; 2 Department of Cardiology, Memorial Bahcelievler Hospital, 34180 Istanbul, Turkey; 3 Department of Cardiology, Prof. Dr. Cemil Tascıoğlu City Hospital, 34384 Istanbul, Turkeykadriye11@yahoo.com (K.K.); 4 Royal Brompton Hospital Guy’s and St Thomas’ NHS Foundation Trust, London SW3 6PY, UK; 5 Department of Cardiology, Gazi University Medical Faculty, 06500 Ankara, Turkey; 6 Department of Biochemistry, Yıldız Teknik University, 34220 Istanbul, Turkey; 7 Department of Biochemistry, Prof. Dr. Suleyman Yalcın City Hospital, 34722 Istanbul, Turkey

**Keywords:** atherosclerosis, complex coronary artery disease, coronary artery disease, homoarginine, SYNTAX score, ST-segment elevation myocardial infarction

## Abstract

**Objective**: Homoarginine is a cationic amino acid derived from lysine. Evidence indicates that low-circulating homoarginine concentration is a risk factor for cardiovascular disease and all-cause mortality. A reduction in homoarginine concentrations has been observed in patients with ischemic heart disease, ischemic stroke, ischemic heart disease, and heart failure. The SYNTAX score (SS), an angiographic scoring system, defines the grade and complexity of coronary artery disease (CAD). The objective of this study was to evaluate the relationship between homoarginine level and the severity of CAD according to the SYNTAX score in patients with ST-segment elevation myocardial infarction (STEMI). **Methods**: A total of 67 subjects were enrolled into the study with the diagnosis of STEMI of those who underwent coronary angiography. STEMI patients were divided into two groups: low-medium SYNTAX score ≤ 14 (35 patients) and high SYNTAX score > 14 (32 patients). **Results**: Within the high SS group, serum homoarginine levels were markedly lower (2 ± 0.9 vs. 1.3 ± 0.7; *p* = 0.001). Homoarginine levels and SS showed a significant negative correlation in entire study cohort In multivariate regression analysis, serum homoarginine levels along with serum urea levels were significantly associated with having higher SS (OR 1.073 *p* = 0.049 and OR 0.346, *p* = 0.012, respectively). **Conclusions**: In conclusion, the diminished plasma homoarginine level emerges as an independent predictor of high atherosclerotic burden among STEMI patients. To the best of our knowledge, this is the first study to demonstrate the relationship between homoarginine and coronary artery complexity.

## 1. Introduction

Cardiovascular diseases (CVD) continue to be a leading cause of global mortality worldwide [1]. Despite the implementations of efficacious measures to control traditional risk factors, a significant number of cardiovascular events still exist [2], indicating the need for further understanding of the pathogenesis of coronary artery disease (CAD) and more effective preventive measures.

Identifying new risk factors for CVD is of utmost importance for improving risk prediction and establishing new treatment targets. Numerous studies have investigated the association between amino acids and CVDs, with increasing recognition of their role in the pathogenesis of CVDs [3]. One such amino acid is homoarginine, a cationic amino acid derived from lysine. Evidence suggests its potential involvement in vasodilator nitric oxide (NO) metabolism [3]. Homoarginine also exhibits other effects related to the cardiovascular system, including the stimulation of insulin secretion and the inhibition of platelet aggregation [4,5]. Clinical evidence indicates that low-circulating homoarginine concentration is a risk factor for CVD and all-cause mortality [6,7]. Reduced homoarginine concentrations have been observed in patients with ischemic heart disease, ischemic stroke, ischemic heart disease, and heart failure [8,9,10].

The SYNergy between percutaneous coronary intervention (PCI) with TAXUS and cardiac surgery (SYNTAX) score is an angiographic scoring system assessing coronary lesion complexity and atherosclerotic burden in patients with CAD [11,12]. It is associated with major adverse cardiac events (MACE) and long-term prognosis of patients undergoing coronary artery bypass grafting (CABG) or percutaneous coronary intervention (PCI) [13,14]. Angiographic data, beyond clinical characteristics, are also utilized to predict long-term prognosis in patients with ST-elevation myocardial infarction (STEMI), a significant component of acute coronary syndromes (ACS) [15,16].

Limited data regarding the association between homoarginine levels and STEMI are available. Currently, no study in the literature investigates the relationship between serum homoarginine levels and STEMI patients undergoing primary PCI. In our study, we aimed to investigate the relationship between plasma homoarginine levels and coronary artery lesion complexity measured by the SYNTAX score in STEMI patients. 

## 2. Materials and Methods

### 2.1. Patient Population and Coronary Intervention

Our study is a prospective cohort and single-center study. A total of 67 consecutive patients who underwent coronary angiography due to ST-segment elevation myocardial infarction (STEMI) were enrolled in the study. Patients were included if they met the following criteria: (1) diagnosis of STEMI with a ST-segment elevation of ≥2 mm in any of the contiguous leads on the electrocardiogram (ECG) and (2) underwent primary percutaneous coronary intervention (PCI) due to a thrombus causing 100% stenosis of one or more coronary arteries in immediate coronary angiography. Patients who met one of the following criteria were excluded: (1) patients with severe heart failure, cardiogenic shock, chronic obstructive pulmonary disease (COPD), liver or kidney dysfunction; (2) patients with acute or chronic infectious or autoimmune diseases or recent use of drugs that have an effect on the immune response; (3) patients who has diagnosed with any kind of malignancy; (4) patients who has a history of coronary artery by-pass grafting surgery and (5) patients who refused to sign the informed consent form and did not want to participate in this research.

Patients with STEMI were admitted within 12 h of symptom onset and blood samples were taken from all patients before the intervention. Detailed demographic and clinical information for all patients was recorded. Complete blood count (CBC) and blood biochemical analyses were conducted, including electrolytes, lipid profile, C-reactive protein (CRP), troponin-T and other routine parameters. Standard-of-care therapy, as per European and American guidelines, was administered, involving aspirin, ticagrelor, or clopidogrel, and unfractionated heparin before PCI. GP IIb/IIIa receptor inhibitors infusions were administered if necessary.

### 2.2. SYNTAX Score and Angiographic Analysis

The SYNTAX score (SS) was calculated utilizing Cardiovascular Angiography Analysis System (CAAS, 5.10, Pie Medical Imaging B.V., Maastricht, The Netherlands) for all patients by two experienced interventional cardiologists on diagnostic angiograms before the PCI. The SS algorithm, which is described in detail elsewhere and available on the SS website (www.syntaxscore.com, accessed on 21 February 2022), was used to retrospectively score all coronary lesions that were considered to have a percentage diameter stenosis of 50% or more in vessels that were 1.5 mm or larger. In the event of a disagreement, a third analyst was consulted, and a final decision was reached through consensus. Patients who had previously undergone CABG operation were excluded from the analysis.

Enrolled individuals were categorized into two groups based on their SS (>14, *n* = 32, and ≤14, *n* = 35). A SS of 14 and below was classified as low-medium and above 14 as high SS. Serum homoarginine levels were compared between these groups.

### 2.3. Homoarginine Detection and Sample Preparation Method

Pre-interventional blood samples (5 mL) were meticulously collected using EDTA tubes, followed by plasma separation through centrifugation at 1000× *g* for 10 min. The resultant plasma samples were diligently preserved at −80 °C until subsequent analysis.

A serum sample of 50 µL was combined with 25 µL of an internal standard solution (comprising 1.5 µM ADMA-d7 and 25 µM Arginine-13C6; 15N4) prepared in 0.1 M HCl. Following this, 150 µL of acetonitrile was added to induce protein precipitation. After vigorous vortex mixing for 1 min, the resulting mixture was incubated at 2–8 °C for 10 min. Subsequently, centrifugation at 8000 rpm for 5 min facilitated the separation of the supernatant, which was then transferred to a vial suitable for LC-MS/MS analysis.

The prepared plasma samples underwent analysis using a validated method on a Shimadzu 8040 LC-MS/MS system coupled with a Fortis HILIC column (50 × 2.00 mm, 5 µm). Gradient method was employed for the chromatographic separation, utilizing a mobile phase consisting of either 100 mM ammonium formate (pH: 3.2) and acetonitrile (800:200 *v*/*v*) or mixtures of acetonitrile, 25 mM ammonium formate (pH: 3.2), and formic acid (800:200:3 *v*/*v*/*v*).

### 2.4. Statistics

Continuous variables were represented as mean ± standard deviation or median and inter-quartile range depending on the normality of distribution, while categorical data were depicted as percentages or frequencies. Normality of distribution for continuous variables was assessed using the Kolmogorov–Smirnov test. The patient cohort was stratified based on SS, with those scoring > 14 constituting a group of 32 individuals and those scoring ≤ 14 comprising a group of 35 individuals. Baseline characteristics were compared between these groups utilizing either the Student’s *t*-test or the χ2 test. Pearson correlation coefficients were employed to elucidate the relationship between SS and homoarginine levels. Conventional clinical variables such as age and gender, along with contributors exhibiting significant correlations, were included in both univariate and multivariate logistic regression analyses to explore predictors associated with elevated SS (>14). A significance threshold of ≤0.05, two-tailed, was accepted for statistical inference. Statistical analyses were performed using SPSS version 25.0 (IBM, Armonk, NY, USA). Graphics were created using MedCalc^®^ Statistical Software (version 20.015; MedCalc Software Ltd., Ostend, Belgium).

## 3. Results

In the cohort of STEMI patients, stratification based on the SS included 67 individuals (mean age: 58.3 ± 9.7 years; 9% female) who underwent PCI, as detailed in Table 1. The clinical, demographic, and laboratory characteristics of the patients are presented in Table 1. Patients were classified into non-high (low and mid) SS groups (*n* = 35, SS ≤ 14) and a high SS group (*n* = 32, SS > 14). The diabetes rate was found to be statistically significant in the high-SS group (*p*-value 0.039), while no statistically significant differences were observed in other patient characteristics. In the high SS group, serum fasting glucose, basal troponin-T levels, and urea values were significantly elevated, while no significant differences were observed in the remaining laboratory parameters (Table 1). A comparison of the echocardiographic findings between the two groups revealed no significant differences, as shown in Table 2.

**Figure 1:**
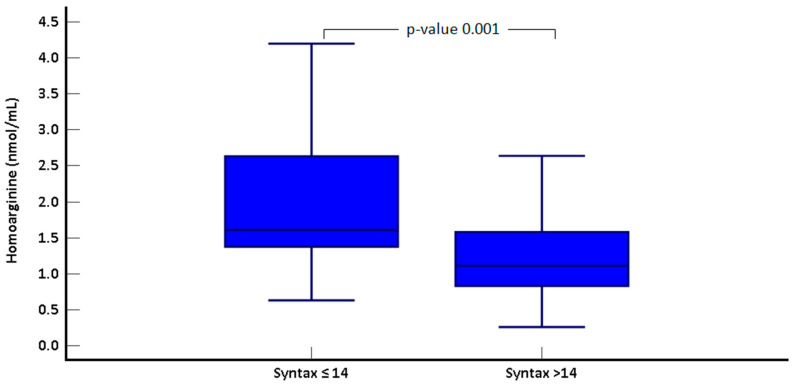
Comparison of Homoarginine levels by Syntax score groups.

**Figure 2:**
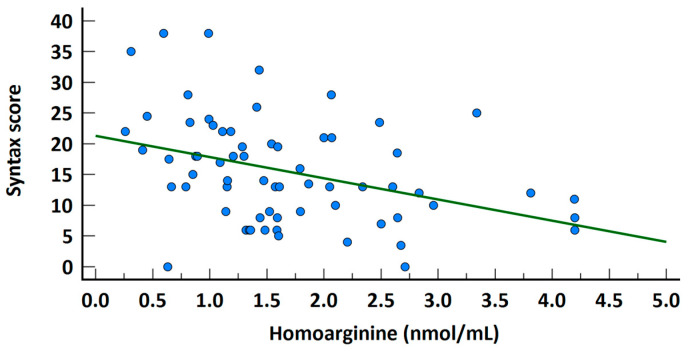
Correlation graph of Homoarginine levels and Syntax score.

In the high SS group, serum homoarginine levels were significantly lower (2 ± 0.9 vs. 1.3 ± 0.7; *p* = 0.001, Figure 1). A significant negative correlation was observed between homoarginine levels and SS across the entire study cohort (rho = −0.375, *p* = 0.002, Figure 2). Univariate regression analysis demonstrated that serum homoarginine levels, along with hypertension, diabetes, and urea levels, were significantly associated with elevated SS (Table 3).

**Figure 3:**
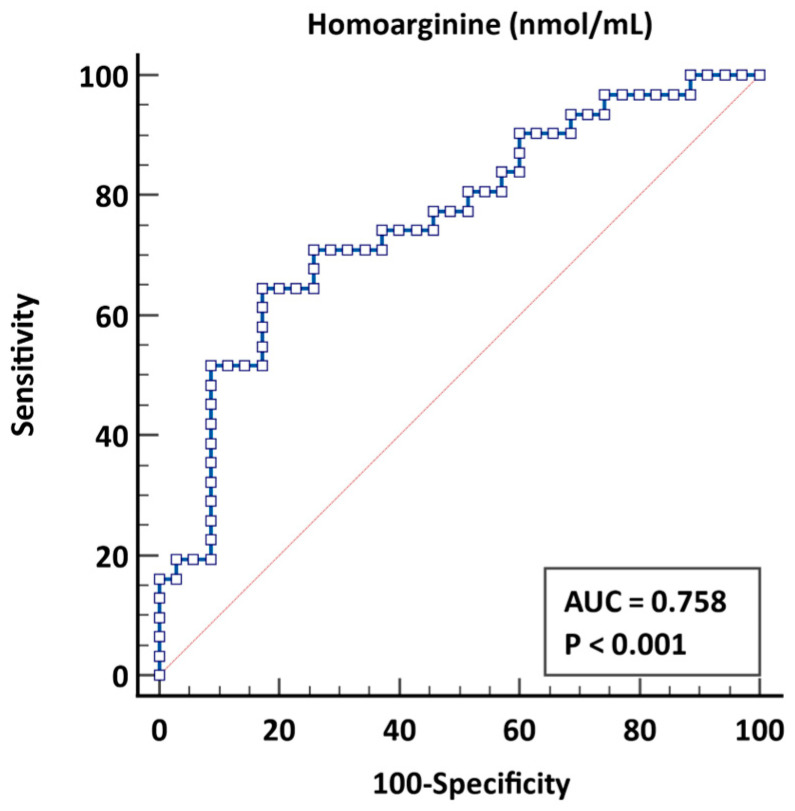
ROC curve for Syntax score > 14.

Furthermore, multivariate regression analysis identified serum homoarginine levels and urea concentrations as independent predictors of higher SS (OR 1.073 *p* = 0.049 and OR 0.346, *p* = 0.012, respectively). Receiver operating characteristic analysis indicated that homoarginine levels ≤ 1.433 could predict a high SS with a sensitivity of 70.97% and a specificity of 74.29% (AUC 0.758, 95% CI 0.639–0.876, *p* < 0.001, as shown in Figure 3).

## 4. Discussion

This study evaluated the prospective relationship between serum homoarginine levels and the SYNTAX score in STEMI patients. The results indicated that low homoarginine levels were an independent predictor of a higher SS in STEMI patients after adjustments for traditional risk factors. Patients with lower homoarginine levels exhibited higher SS, indicating more severe coronary artery atherosclerosis.

Despite advances in reperfusion therapy and secondary prevention, STEMI remains a leading cause of early mortality worldwide [15]. The atherosclerotic burden in CAD is independently associated with increased mortality, and angiographic data, such as the number of diseased coronary arteries, are used to predict long-term prognosis [16]. Several systems have been established to evaluate atherosclerotic burden, determine the extent of atherosclerosis, and provide prognostic information [17,18]. The SYNTAX Score (SS) is an angiographic tool that evaluates the extent and complexity of CAD, with higher scores correlating with worse outcomes and increased major cardiovascular events in PCI patients [11,19]. Initially used to guide revascularization strategies in stable CAD, the SS has recently been applied to STEMI patients undergoing primary PCI [20]. In a study by Girasis et al., patients were stratified into high and non-high SS groups [21]. Previous studies, including those by Garg et al., Biondi-Zoccai et al., and Brown et al., have highlighted the prognostic value of the SS in predicting adverse outcomes such as MACE, mortality, and stent thrombosis [13,14,22].

The pathogenesis and progression of atherosclerotic lesions, which refers to the increased atherosclerotic burden, is a complex process involving multiple inflammatory factors. This ultimately may lead to plaque rupture and myocardial infarction [23,24]. Several recent studies suggest that the relative presence and absence of certain amino acids may contribute to the development and occurrence of atherosclerosis. For instance, elevated circulating concentrations of homocysteine and free asymmetric dimethylarginine are long-established cardiovascular risk factors among these amino acids [25]. Another amino acid is homoarginine, a homolog of L-arginine, acts as a cationic amino acid that can enhance nitric oxide (NO) production by inhibiting arginase and protein arginine methyltransferases, serving as a substrate for NO synthases and improving endothelial function and its importance as a cardiovascular risk factor is increasing [26,27]. Disruption of NO metabolism and decreased bioactivity leads to endothelial dysfunction, a critical point in atherogenesis [28].

While the exact mechanism underlying the association of homoarginine with cardiovascular risk remains incompletely understood, its primary role in NO formation is emphasized. Homoarginine functions as both a substrate and a weak inhibitor of arginase, thereby increasing the availability of the NO precursor L-arginine [29].

Decreased homoarginine levels have been observed in patients with renal, cardiovascular, and cerebrovascular diseases. In one study, low-circulating homoarginine concentrations were reported as prognostic markers for mortality and cardiovascular events in various diseases including heart failure, stroke, and chronic kidney disease [3]. Increasing evidence suggests that low homoarginine levels act as independent risk factor for cardiovascular events and is directly related to cardiovascular mortality [25]. A study involving 3305 patients undergoing coronary angiography demonstrated that low homoarginine concentrations were associated with impaired left ventricular function and increased risk of mortal events. One of the study’s main findings was the identification of low serum homoarginine levels as a new biomarker for heart failure [30]. Experimental studies have also demonstrated exogenous homoarginine’s anti-hypertensive and antithrombotic effects [5,31].

In the Dallas Heart Study, the relationship between plasma homoarginine levels and clinical outcomes was evaluated in 3514 patients from multiple ethnic groups, identifying an independent inverse relationship between circulating homoarginine levels and MACE and all-cause mortality [29]. Similarly, plasma homoarginine concentrations were measured during the 6th follow-up visit in the Framingham Offspring Study, and correlations with cardiovascular disease and mortality were evaluated. Lower circulating homoarginine levels were found to be associated with a higher risk of death over a follow-up period of more than 18 years, even after adjusting for known cardiovascular risk factors [32].

To the best of our knowledge, there is currently no study in the literature investigating homoarginine levels in patients with isolated STEMI. However, in a study involving 1649 patients presenting with acute chest pain, 589 were diagnosed with ACS and during the average 183-day follow-up period, 60 MACE, including all-cause mortality, stroke or myocardial infarction were recorded in the overall study population, with 43 MACE observed in the ACS subgroup. According to the findings of this study, low plasma homoarginine is a risk marker for MACE, particularly in patients with acute chest pain, especially those with elevated hs-Troponin-I levels. Additionally, impaired homoarginine levels were found to be associated with prevalent atrial fibrillation [9].

Considering the association between the SS, the most widely used anatomical scoring system globally, and the prognosis of STEMI patients, as well as the fact that the severity of CAD is linked to an increased risk of cardiovascular mortality, it is possible to conclude that patients with STEMI and lower homoarginine levels at presentation are at a higher risk of cardiovascular morbidity and mortality.

## 5. Conclusions

In conclusion, the diminished plasma homoarginine level emerges as an independent predictor of high atherosclerotic burden among STEMI patients. Further investigations into the underlying mechanisms through which reduced levels contribute to coronary atherosclerosis, as well as the utility of homoarginine levels in risk stratification for CAD patients, are imperative.

Additional investigations into the pathways implicated in the diminished homoarginine levels leading to escalated atherosclerotic burden are warranted. Moreover, it remains to be elucidated whether homoarginine could serve as a viable therapeutic target for the prevention and treatment of atherosclerosis.

## 6. Limitations

Our study is a single-center prospective study with a relatively limited number of patients. A secondary constraint is the absence of an age-matched control group. Moreover, the homogeneity of the study population in terms of race and ethnicity suggests that the findings may not be generalizable to more diverse populations. Another limitation is the non-fasting blood samples during the acute phase of MI, thereby precluding the exclusion of alternative sources of NO linked to dietary factors.

## Figures and Tables

**Table 1:** Basic biochemical and clinical features of the patient groups.

	Syntax ≤ 14 (*n* = 35)	Syntax > 14 (*n* = 32)	*p*-Value
Age (year)	57.2 ± 8.8	59.6 ± 10.6	0.322
Female Gender (*n*, %)	3 (8.6)	3 (9.4)	0.999
Male Gender (*n*, %)	32 (91.4)	29 (90.6)	0.908
Hypertension (*n*, %)	11 (31.4)	18 (56.3)	0.051
Diabetes (*n*, %)	4 (11.4)	11 (34.4)	0.039 *
Current smoker (*n*, %)	22 (62.9)	16 (50)	0.331
Height (cm)	168.6 ± 6.8	169.3 ± 6.9	0.697
Weight (kg)	73.6 ± 9.4	71.9 ± 6.9	0.408
Body mass index (kg/m^2^)	25.2 ± 2.6	24.5 ± 2	0.239
Serum fasting glucose (mg/dL)	110 ± 28.4	144.9 ± 67.1	0.007 *
Hemoglobin (g/dL)	13.5 ± 2.2	12.8 ± 2.8	0.216
WBC count (10^3^ μL)	10.8 ± 4.5	11.6 ± 4.6	0.482
Creatinine (mg/dL)	0.9 ± 0.2	0.93 ± 0.3	0.615
GFR (mL/min per 1.73 m^2^)	90.5 ± 17.2	83 ± 24.3	0.153
ALT (U/L)	25.4 ± 14.8	36.6 ± 35	0.090
AST (U/L)	52.1 ± 77.7	63.3 ± 61	0.519
Total cholesterol (mg/dL)	182.9 ± 45.9	176.7 ± 48.6	0.605
LDL cholesterol (mg/dL)	116.1 ± 41	109.4 ± 34.3	0.478
HDL cholesterol (mg/dL)	37.6 ± 7.9	39 ± 9.8	0.533
Triglyceride (mg/dL)	171.1 ± 71.1	166.4 ± 93.2	0.820
Troponin-T (ng/L) (basal)	52.3 ± 85.6	265.8 ± 581.1	0.035 *
Troponin-T (ng/L) (peak)	1905.6 ± 2240	2598.5 ± 2660.5	0.252
Urea mg/dL	33.03 ± 10.9	43 ± 16.7	0.006 *
Homoarginine(nmol/mL)	2 ± 0.9	1.3 ± 0.7	0.001 *

* statistically significant. Abbreviations: FBG: fasting blood glucose, WBC: white blood cell, GFR: glomerular filtration rate, ALT: alanine transaminase, AST: aspartate transaminase, LDL: low-density lipoprotein, HDL: high-density lipoprotein.

**Table 2:** Angiographic and echocardiographic features of patients group.

	Syntax ≤ 14 (*n* = 35)	Syntax > 14 (*n* = 32)	*p*-Value
Single vessel	18 (51.4)	3 (9.4)	<0.001 *
Two-vessel	15 (42.9)	14 (43.8)	<0.001 *
Three- vessel	2 (5.7)	15 (46.9)	<0.001 *
LA (cm)	3.7 ± 0.5	3.8 ± 0.4	0.299
LVEDd (cm)	4.9 ± 0.4	4.8 ± 0.4	0.383
LVESd (cm)	3.4 ± 0.5	3.4 ± 0.6	0.948
RVEDd (cm)	2.8 ± 0.5	2.8 ± 0.4	0.972
RA (cm)	3.4 ± 0.3	3.5 ± 0.4	0.195
TAPSE (cm)	2 ± 0.1	2 ± 0.2	0.270
LVEF (%)	51.1 ± 8.9	49.4 ± 10.9	0.514
sPAP (mmHg)	23.2 ± 6.2	24.8 ± 6.7	0.339

* statistically significant. Abbreviations: LVEF: Left ventricular ejection fraction, LVEDd: Left ventricular end-diastolic diameter, LVESd: Left ventricular end-systolic diameter, LA: Left atrium, RA: Right atrium, RVEDd: Right ventricular end-diastolic diameter, TAPSE: Tricuspid annular plane systolic excursion, sPAP: Systolic pulmonary artery pressure.

**Table 3:** Univariate and Multivariate Regression Analysis for Syntax > 14.

	Univariate				Multivariate			
Variables			95% CI				95% CI	
	OR	*p*-Value	Lower	Upper	OR	*p*-Value	Lower	Upper
Female sex	1.103	0.908	0.206	5.905				
Age	1.026	0.318	0.975	1.080				
Smoking	0.591	0.290	0.223	1.566				
Hypertension	2.805	0.043 *	1.033	7.614	1.153	0.821	0.335	3.963
Diabetes	4.060	0.031 *	1.138	14.475	0.565	0.548	0.088	3.633
Family history	1.493	0.434	0.547	4.072				
Urea	1.066	0.010 *	1.016	1.119	1.073	0.049 *	1.000	1.151
Homoarginine	0.313	0.003 *	0.146	0.671	0.346	0.012 *	0.151	0.792

* statistically significant.

## Data Availability

The data presented in this study are available on request from the corresponding author due to privacy and ethical reasons.

## Abbreviations

The following abbreviations are used in this manuscript:

SYNTAX	The SYNergy between percutaneous coronary intervention (PCI) with TAXUS and cardiac surgery
CAD	Coronary artery disease
STEMI	ST-segment elevation myocardial infarction
NO	Nitric oxide
ACS	Acute coronary syndrome
MI	Myocardial infarction
SS	SYNTAX score
CABG	Coronary artery by-pass grafting
CVD	Cardiovascular disease
MACE	Major adverse cardiac events
COPD	Chronic obstructive pulmonary disease
